# Supplementary material for: Look what you make my tissues do: The role of metalloproteinases and their inhibitors in Bothrops snakebites
Source: PLoS Negl Trop Dis. 2026 Apr 24;20(4):e0013831. doi: 10.1371/journal.pntd.0013831 (PMC13128130; doi:10.1371/journal.pntd.0013831)
Supplement: S1 Table — (DOCX) [file pntd.0013831.s001.docx]

**Supplementary Table 1.** Circulating levels of MMPs and TIMPs in healthy donors and patients with mild or severe suspected *Bothrops* envenomation at baseline (T0).

| **Marker** | **HD**  (n=20) | **Mild**  (n=15) | **Severe**  (n=15) | ***p-value***  ***HD vs Mild*** | ***p-value***  ***HD vs Severe*** | ***p-value***  ***Mild vs Severe*** |
| --- | --- | --- | --- | --- | --- | --- |
| **Metalloproteinases** | | | | | | |
| **MMP-1**, median (IQR) | 134.7  (93.5-309.7) | 214.0  (95.4-305.3) | 239.2  (95.9-819.6) | 0.268 | 0.202 | 0.389 |
| **MMP-2**, median (IQR) | 952.5  (808.3-1147.0) | 1019.0  (481.2-1638.0) | 961.2  (326.7-1657.0) | 0.631 | 0.897 | 0.837 |
| **MMP-7**, median (IQR) | 2559.0  (2368.0-4212.0) | 6009.0  (2791.0-9527.0) | 4869.0  (2929.0-7800.0) | 0.902 | **0.0045** | 0.925 |
| **MMP-9**, median (IQR) | 6292.0  (3639.0-8614.0) | 1424.0  (652.3-5928.0) | 3340.0  (887.0-7199.0) | **0.0001** | **0.0167** | 0.137 |
| **MMP-10**, median (IQR) | 132.5  (75.7-161.0) | 143.3  (121.8-326.5) | 204.0  (109.8-311.8) | **0.0336** | **0.0004** | 0.511 |
| **Tissue Inhibitors of Metalloproteinases** | | | | | | |
| **TIMP-1**, median (IQR) | 492.4  (455.3-560.2) | 947.4  (820.0-1218.0) | 1050.0  (886.1-1471.0) | **0.0001** | **0.0001** | 0.412 |
| **TIMP-2**, median (IQR) | 763.4  (698.9-903.6) | 1121.0  (983.0-1347.0) | 1521  (1110.0-2003.0) | **0.0001** | **0.0006** | 0.743 |
| **TIMP-3**, median (IQR) | 482.0  (394.3-612.9) | 757.4  (420.4-1049.0) | 957.1  (518.7-1071.0) | 0.075 | **0.0034** | 0.623 |
| **TIMP-4**, median (IQR) | 147.8  (107.2-186.8) | 195.2  (125.0-444.4) | 329.9  (118.6-467.3) | **0.0399** | **0.0084** | 0.623 |

Data are presented as median and interquartile range (IQR). HD: Healthy Donors. Statistical analysis was performed using the Kruskal-Wallis test followed by Dunn’s post-test. **Statistically significant p-values are highlighted in bold (p<0.05)**
