# Supplementary material for: Look what you make my tissues do: The role of metalloproteinases and their inhibitors in Bothrops snakebites
Source: PLoS Negl Trop Dis. 2026 Apr 24;20(4):e0013831. doi: 10.1371/journal.pntd.0013831 (PMC13128130; doi:10.1371/journal.pntd.0013831)
Supplement: S2 Table — (DOCX) [file pntd.0013831.s002.docx]

**Supplementary Table 2.** Longitudinal changes in circulating MMP and TIMP levels in patients with Mild and Severe suspected *Bothrops* envenomation following antivenom administration.

| **Marker** | **HD**  (n=20) | **Mild T0**  n=15 | **Mild T1**  n=15 | **Mild T2**  n=15 | **Severe T0**  n=15 | **Severe T1**  n=15 | **Severe T2**  n=15 |
| --- | --- | --- | --- | --- | --- | --- | --- |
| **Metalloproteinases** | | | | | | |  |
| **MMP-1**, median (IQR) | 134.7  (93.49-309.7) | **214.0^*^**  **(95.4-305.3)** | 121.8  (80.4-233.1) | **219.7**^*^**^c^**  **(106.5-363.0)** | **239.2^*^**  **(95.9-819.6)** | **130.9^a^**  **(64.0-192.3)** | **95.0^b#^**  **(50.4-162.9)** |
| **MMP-2**, median (IQR) | 952.5  (808.3-1147.0) | 1019.0  (481.2-1638.0) | **750.8^a^**  **(257.2-1139.0)** | **697.9^*b^**  **(574.2-934.8)** | 961.2  (326.7-1657.0) | **964.5^*a^**  **(418.1-852.1)** | **574.2^*b#^**  **(235.3-584.6)** |
| **MMP-7**, median (IQR) | 2559.0  (2368.0-4212.0) | **6009.0^*^**  **(2791.0-9527.0)** | 3666.0  (2403.0- 6924.0) | **5512.0^*^**  **(2941.0- 8512.0)** | **4869.0^*^**  **(2929.0-7800.0)** | **3552.0^*a^**  **(2639.0-7577.0)** | **2989.0^b#^**  **(1978.0-3833.0)** |
| **MMP-9**, median (IQR) | 6292.0  (3639.0-8614.0) | **1424.0^*^**  **(652.3-5928.0)** | **422.0^*a^**  **(221.4-2217.0)** | **452.6^*b^**  **(264.0-1987.0)** | **3340.0^*^**  **(887.0-7199.0)** | **641.6^*a^**  **(301.4-1927.0)** | **131.5^*b,c#^**  **(89.5-208.9)** |
| **MMP-10**, median (IQR) | 132.5  (75.67-161) | **143.3^*^**  **(121.8-326.5)** | **118.1^a^**  **(77.8-201.9)** | 186.8  (110.6-315.6) | **204.0^*^**  **(109.8-311.8)** | **101.5^a^**  **(59.6-128.3)** | **114.6^b#^**  **(68.8-178.7)** |
| **Tissue Inhibitors of Metalloproteinases** | | | | | | |  |
| **TIMP-1**, median (IQR) | 492.4  (455.3-560.2) | **947.4^*^**  **(820.0-1218.0)** | **817.9^*a^**  **(726.3-890.8)** | **845.0^*b^**  **(767.3-930.3)** | **1050.0^*^**  **(886.1-1471.0)** | **794.7^*a^**  **(744.8-949.7)** | **835.1^*b^**  **(782.1-923.7)** |
| **TIMP-2**, median (IQR) | 763.4  (698.9-903.6) | **1121.0^*^**  **(983.0-1347.0)** | **955.8^*a^**  **(919.2-996.4)** | **988.7^*b^**  **(842.0-1076.0)** | **1521.0^*#^**  **(1110.0-2003.0)** | **943.9^*a^**  **(802.2-1057.0)** | **985.3^*b^**  **(916.9-1080.0)** |
| **TIMP-3**, median (IQR) | 482.0  (394.3-612.9) | **757.4^*^**  **(420.4-1049.0)** | 667.3  (540.7-952.5) | **610.4^b^**  **(438.3-646.8)** | **957.1^*^**  **(518.7-1071.0)** | **786.7^*^**  **(662.2-834.9)** | **573.3^b,c^**  **(426.4-687.7)** |
| **TIMP-4**, median (IQR) | 147.8  (107.2-186.8) | **195.2^*^**  **(125.0-444.4)** | **518.0^*a^**  **(229.7-614.7)** | **165.1^*b^**  **(112.6-201.4)** | **329.9^*^**  **(118.6-467.3)** | **480.5^*a^**  **(224.1-584.0)** | **168.3^*b^**  **(103.4-249.5)** |

Data are presented as median and interquartile range (IQR). Statistical analysis was performed using the Kruskal-Wallis test followed by Dunn’s post-test. Significant differences compared to the HD group are highlighted with asterisks (*). Significant differences between the T0 and T1 groups are represented by the letter “a”; Significant differences between the T0 and T2 groups are represented by the letter “b”; Significant differences between the T1 and T2 groups are represented by the letter “c”; Significant differences between the Mild and Severe groups are represented by the hash symbol (#). **Statistically significant p-values are highlighted in bold (p<0.05).**
