# Supplementary material for: Look what you make my tissues do: The role of metalloproteinases and their inhibitors in Bothrops snakebites
Source: PLoS Negl Trop Dis. 2026 Apr 24;20(4):e0013831. doi: 10.1371/journal.pntd.0013831 (PMC13128130; doi:10.1371/journal.pntd.0013831)
Supplement: Attachment — Submitted filename: Response Letter to Reviewers.docx [file pntd.0013831.s003.docx]

**Response Letter to Reviewers**

**Manaus, AM, Brazil, February 2nd, 2026**

**Dear Editors,**

**Hatem Kallel
Academic Editor**

**José María Gutiérrez
Section Editor

Shaden Kamhawi
co-Editor-in-Chief**

We appreciate the opportunity to revise our manuscript by Neves *et al*. (ID: PNTD-D-25-02175), ***Look What You Make My Tissues Do: The Role of Metalloproteinases and Their Inhibitors in Bothrops Snakebites*** at PLOS Neglected Tropical Diseases.

We have carefully addressed all the comments and suggestions provided, and we believe the revisions have significantly strengthened the manuscript. We are hopeful that the revised version now meets the standards of the journal. Below, we provide a detailed point-by-point response to the comments and questions raised by the reviewers and editors. Revisions to the manuscript are highlighted in yellow throughout the text.

Once again, we sincerely thank the reviewers for their thoughtful input and the editorial team for considering our work for publication.

Sincerely,

Allyson Guimarães Costa

Corresponding author

(On behalf of all co-authors)

**JOURNAL REQUIREMENTS**

***1) Please ensure that the CRediT author contributions listed for every co-author are completed accurately and in full.***

***At this stage, the following Authors/Authors require contributions: Juliana Costa Ferreira Neves, Fábio Magalhães-Gama, Hiochelson Najibe Santos Ibiapina, Kamille Beltrão Seixas, Êndila Souza Barbosa, Adriana Malheiro, Jacqueline de Almeida Gonçalves Sachett, Ana Carolina Campi-Azevedo, Olindo Assis Martins-Filho, Andréa Teixeira-Carvalho, Marco Aurélio Sartim, Wuelton Monteiro, and Allyson Guimarães Costa. Please ensure that the full contributions of each author are acknowledged in the "Add/Edit/Remove Authors" section of our submission form.***

We thank the Editorial Office for the reminder. We have now completed the CRediT author contribution roles for all co-authors in the “Add/Edit/Remove Authors” section of the submission system, ensuring that each author’s contributions are accurately and fully reported according to the PLOS CRediT taxonomy.

***2) We ask that a manuscript source file is provided at Revision. Please upload your manuscript file as a .doc, .docx, .rtf or .tex. If you are providing a .tex file, please upload it under the item type u2018LaTeX Source Fileu2019 and leave your .pdf version as the item type u2018Manuscriptu2019.***

As requested, we have uploaded the revised manuscript source file in an editable format (.docx) under the item type “Manuscript Source File,” while keeping the PDF under the item type “Manuscript.”

***3) Please ensure that the Title in your manuscript file and the Title provided in your online submission form are the same.***

We have carefully checked and confirmed that the title provided in the manuscript file and the title entered in the online submission system are identical.

***4) We do not publish any copyright or trademark symbols that usually accompany proprietary names, eg ©,  ®, or TM  (e.g. next to drug or reagent names). Therefore please remove all instances of trademark/copyright symbols throughout the text, including:***

***- ® on page: 4.***

All trademark and copyright symbols (e.g., ®, ™) have been removed throughout the manuscript text, in accordance with PLOS journal guidelines.

***5) Some material included in your submission may be copyrighted. According to PLOSu2019s copyright policy, authors who use figures or other material (e.g., graphics, clipart, maps) from another author or copyright holder must demonstrate or obtain permission to publish this material under the Creative Commons Attribution 4.0 International (CC BY 4.0) License used by PLOS journals. Please closely review the details of PLOSu2019s copyright requirements here: PLOS Licenses and Copyright. If you need to request permissions from a copyright holder, you may use PLOS's Copyright Content Permission form.***

***Please respond directly to this email and provide any known details concerning your material's license terms and permissions required for reuse, even if you have not yet obtained copyright permissions or are unsure of your material's copyright compatibility. Once you have responded and addressed all other outstanding technical requirements, you may resubmit your manuscript within Editorial Manager.***

Figures 1 and 6 were created using BioRender.com under a paid academic license. BioRender grants authors the right to publish these figures in academic journals. We confirm that the figures comply with PLOS licensing requirements, and the following statement has been included in the figure captions: “Created with BioRender.com.”

***6) Please provide a complete Data Availability Statement in the submission form, ensuring you include all necessary access information or a reason for why you are unable to make your data freely accessible. If your research concerns only data provided within your submission, please write "All data are in the manuscript and/or supporting information files" as your Data Availability Statement.***

We have revised the Data Availability Statement to fully comply with PLOS data policy. The statement now clearly explains the ethical restrictions related to patient privacy, provides the relevant ethics approval information, and specifies the conditions under which de-identified data may be accessed.

***7) Please send a completed 'Competing Interests' statement, including any COIs declared by your co-authors. If you have no competing interests to declare, please state "The authors have declared that no competing interests exist". Otherwise please declare all competing interests beginning with the statement "I have read the journal's policy and the authors of this manuscript have the following competing interests"***

A completed Competing Interests statement has been provided. The authors declare that no competing interests exist.

**REVIEW 1**

***1) 2.3 – duplication (with minor differences) of the first two paragraphs. How was Bothrops envenoming identified? This isn’t described, and if no formal diagnosis, edit to “suspected Bothrops sp.”***

We thank the reviewer for this observation. The duplicated text in Section 2.3 has been removed. We have also clarified how cases were identified and now consistently refer to them as suspected Bothrops spp. envenomation, based on clinical and epidemiological criteria in accordance with Brazilian Ministry of Health guidelines.

***2) 2.3 - It would be helpful to contextualise the T0 timepoint. Is this shortly after admission? How much variation was there in terms of time from bite to T0 – please provide a range.***

We have revised Section 2.3 to clearly contextualize the T0 time point. T0 is now explicitly defined as blood collection performed immediately after hospital admission and prior to antivenom administration, as part of the initial clinical evaluation. This clarification addresses the timing of sample collection relative to patient admission and treatment initiation.

***3) 2.4 – This section needs information on replicates, both in terms of technical and independent replicates, where applicable.***

We thank the reviewer for this important suggestion. Section 2.4 has been revised to explicitly describe both biological and technical replicates. Each patient sample was considered an independent biological replicate, and all measurements were performed in duplicate as technical replicates, with the mean values used for statistical analyses.

***4) 2.6 – What was the rationale for using median of the three groups as the cutoff value rather than the mean (or mean + a factor of the standard deviation) of the HD control?***

We appreciate this methodological question. The global median across Healthy Donor, Mild, and Severe groups was intentionally selected as the cutoff value to reduce the influence of outliers and non-Gaussian distributions frequently observed in clinical biomarker data. This strategy allows direct comparison of relative expression patterns across all study groups and is appropriate for the exploratory nature of the present analysis, rather than aiming to establish absolute clinical thresholds.

***5) Figure 3 – significant differences needs to be better shown. This might be currently hampered by the figure resolution.***

We thank the reviewer for this observation. Figure 3 has been revised to improve the clarity and visualization of statistically significant differences. All figures in the manuscript were regenerated in high resolution using the PLOS NAAS tool and GIMP software, and font sizes, axis labels, and statistical symbols were adjusted to enhance readability.

We note that the submitted figure files are available to reviewers and editors in high-resolution format when accessed directly, as opposed to the compressed resolution typically displayed in the PDF version generated during submission. These revisions ensure that the figure can be viewed with excellent resolution and quality when accessed as individual figure files.

***6) More discussion of the limitations are required.
Much of the discussion contains assertions based on predictions of the visualised correlations, and I think the authors would be better placed to reword these sections to highlight that this study provides valuable pilot data that will enable them (or others) to more robustly test explicit hypotheses in future studies with larger sample sizes.***

We thank the reviewer for this important and constructive comment. The Discussion has been revised to more explicitly acknowledge the limitations of the study, including the relatively small sample size and the exploratory nature of the correlation-based and network analyses. We have reworded relevant sections to emphasize that the findings should be interpreted as hypothesis-generating pilot data rather than evidence of causal mechanisms. In addition, we now more clearly highlight that these results provide a framework for future studies with larger cohorts and expanded immunological profiling to robustly test the hypotheses proposed here.

***7) Overall this is an interesting study, which is pretty novel for the field of snakebite. The authors quantify key MMPs and TIMPs from clinical samples collected from envenomed patients in the Brazilian Amazon and interrogate the resulting dataset in the context of mild and severe stratified patients. Further elaboration of the methods is required, and I think a more robust discussion section that draws more widely on the literature, further highlights limitations, and perhaps explains what key next steps are required to validate the hypotheses generated from this data, would make the paper more impactful to the field.***

We thank the reviewer for the positive and encouraging assessment of our study and for highlighting its novelty and relevance to the field of snakebite research. In response to these comments, we have further elaborated the Methods section to improve clarity and transparency, including additional details on case classification, sampling procedures, replicates, and data analysis strategies.

In addition, the Discussion has been substantially revised to draw more broadly on the existing literature, more explicitly acknowledge the limitations of the study, and emphasize the exploratory and hypothesis-generating nature of the findings. We now discuss how the observed MMP/TIMP patterns should be interpreted as preliminary evidence and outline key next steps for future investigations, including validation in larger cohorts and the integration of additional inflammatory and venom-related mediators.

We believe that these revisions strengthen the manuscript and enhance its impact by clearly positioning the present work as a foundation for future mechanistic and translational studies in Bothrops envenomation.

**REVIEW 2**

***1) The graphics are mostly low-resolution, making it impossible to read the values on the Y axis.***

***I suggest having a supplementary table containing the raw values per experiment.***

We thank the reviewer for this helpful suggestion. All figures in the manuscript were regenerated in high resolution using the PLOS NAAS tool and GIMP software, with improved readability of axes and labels.

In addition, to enhance data transparency, we have included two supplementary tables providing the numerical values underlying the graphical representations. Supplementary Table S1 corresponds to Figure 2 and presents baseline MMP and TIMP levels across study groups, while Supplementary Table S2 corresponds to Figure 3 and details longitudinal changes following antivenom administration. These additions facilitate interpretation of the results and address concerns related to figure resolution.

***2) I consider that the conclusions are just partially supported by the data presented. As explained in the general comments, the variation in MMP/TIMP appears subtle and should be complemented by investigating additional parameters in the individuals.***

We thank the reviewer for this important and thoughtful comment. We agree that the observed variations in MMP and TIMP levels are relatively subtle and that the conclusions should be interpreted with appropriate caution. Accordingly, we have revised the Discussion and Conclusion sections to more clearly emphasize the exploratory and hypothesis-generating nature of the findings.

We now explicitly acknowledge that additional inflammatory and venom-related parameters were not assessed in the present study and that future investigations incorporating broader immunological profiling and larger cohorts will be required to validate and extend the hypotheses proposed here.

***3) Note, that on lines 119-129 there are duplicated information on both paragraphs***

We thank the reviewer for noting this duplication. The repeated information in lines 119–129 has been removed, and the text has been revised to eliminate redundancy and improve clarity.

***4) The article addresses an important aspect of snake envenomation: the secondary activation of endogenous proteinases, in this case MMPs, which may exacerbate its effects or mediate recovery. The experimental approach was based on measuring MMP and TIMPs (MMP-inhibitors) in healthy or envenomated human subjects (treated or not with the antiserum) using standard methodologies. No other mediators or specific pathologic effects were evaluated.***

***The results indicate a discrete alteration in MMP and TIMP levels upon envenoming, slightly associated with the level of severity. Most of the comparisons between cohorts were shown to be statistically significant according to the tests applied. However, the values indicated in the graphs seem to be generally very similar among the groups and variables within samples. Since the study is based on various clinical samples, especially from real bite accidents that may vary in effectiveness, amount of venom injected, etc., it is expected to obtain such heterogeneous values per group. However, with such minor variation in MMP and TIMP, and the absence of information about other mediators, it is very hard to interpret the results and draw the inflammation model proposed.***

***I suggest presenting the measured values in a separate (supplementary) table and demonstrating the statistical tests performed. And, to better sustain the conclusions about the mechanisms involved uppon MMP activation, other inflammatory indicators should be evaluated.***

***Nevertheless, the article provides strong evidence for the activation of MMPs and the timeline of the secondary steps of envenomation. This is an important contribution to the dynamics of snake envenoming and to the development of complementary treatments.***

We thank the reviewer for this comprehensive and thoughtful evaluation of our manuscript and for recognizing the relevance of investigating secondary activation of endogenous proteinases during snake envenomation. We agree that this study focused specifically on circulating MMPs and their inhibitors, measured using standard methodologies in healthy donors and envenomed patients, and did not assess additional inflammatory mediators or direct pathological endpoints.

We also acknowledge that the observed alterations in MMP and TIMP levels were relatively subtle and that clinical heterogeneity inherent to real-world snakebite events—such as variability in venom dose, injection depth, and time to treatment—likely contributes to overlapping values across groups. Accordingly, we have revised the Discussion and Conclusion sections to adopt a more cautious tone, explicitly emphasizing the exploratory and hypothesis-generating nature of the findings and avoiding mechanistic overinterpretation of correlation-based analyses.

In response to the reviewer’s suggestion to improve data transparency and interpretability, we regenerated all figures in high resolution and included supplementary tables presenting the numerical values underlying Figures 2 and 3, along with the corresponding statistical analyses. These additions allow clearer evaluation of the magnitude and variability of MMP and TIMP levels across groups and time points.

We agree that additional inflammatory and venom-related mediators were not evaluated in the present study and that their inclusion will be essential to more robustly test the inflammatory model proposed. This limitation is now explicitly acknowledged in the revised manuscript, and we outline future directions incorporating broader immunological profiling and larger cohorts to validate and extend the hypotheses generated here.

Finally, we appreciate the reviewer’s recognition that the study provides evidence for MMP activation and its temporal dynamics following envenomation. We believe that, by clearly positioning these results as preliminary and descriptive, the revised manuscript contributes meaningful insight into the secondary molecular events of snake envenoming and provides a foundation for future mechanistic and translational investigations.
