# Supplementary material for: Look what you make my tissues do: The role of metalloproteinases and their inhibitors in Bothrops snakebites
Source: PLoS Negl Trop Dis. 2026 Apr 24;20(4):e0013831. doi: 10.1371/journal.pntd.0013831 (PMC13128130; doi:10.1371/journal.pntd.0013831)
Supplement: Attachment — Submitted filename: Response_Letter_to_Reviewers_auresp_2.docx [file pntd.0013831.s004.docx]

**Response Letter to Reviewers**

**Manaus, AM, Brazil, February 9^th^, 2026**

**Dear Editors,**

**José María Gutiérrez
Section Editor**

**Shaden Kamhawi**

**co-Editor-in-Chief**

**Paul Brindley**

**co-Editor-in-Chief**

We appreciate the opportunity to revise our manuscript by Neves *et al*. (ID: PNTD-D-25-02175), ***Look What You Make My Tissues Do: The Role of Metalloproteinases and Their Inhibitors in Bothrops Snakebites*** at PLOS Neglected Tropical Diseases.

We have carefully addressed all the comments and suggestions provided, and we believe the revisions have significantly strengthened the manuscript. We are hopeful that the revised version now meets the standards of the journal. Below, we provide a detailed point-by-point response to the comments and questions raised by the reviewers and editors. Revisions to the manuscript are highlighted in yellow throughout the text.

Once again, we sincerely thank the reviewers for their thoughtful input and the editorial team for considering our work for publication.

Sincerely,

Allyson Guimarães Costa

Corresponding author

(On behalf of all co-authors)

**EDITOR COMMENTS:**

**1) The reviewers of this manuscript valued the novelty of the study and its relevance to understand the complex dynamics of the inflammatory response to snakebite envenoming in samples from patients. At the same time, they highlighted a number of issues that should be considered for preparing a revised version, including a more detailed description of methods, a refinement of the discussion, with further details about the limitations of the study, and the inclusion of the raw data in a supplementary table. Also, the authors may consider the inclusion of additional inflammatory parameters to make the analysis stronger.**

We agree that additional inflammatory mediators would further strengthen the mechanistic interpretation of our findings. However, due to the limited sample volume obtained from each patient and the real-world clinical setting of this study, we focused on MMPs and TIMPs as key modulators of tissue remodeling. This limitation is now explicitly acknowledged in the revised Discussion, and we emphasize that the present findings are exploratory and hypothesis-generating, providing a foundation for future studies incorporating broader immunological profiling.

**JOURNAL REQUIREMENTS:**

**1) Please ensure that the funders and grant numbers match between the Financial Disclosure field and the Funding Information tab in your submission form. Note that the funders must be provided in the same order in both places as well.**

We confirm that the Financial Disclosure statement has been reviewed and updated to ensure full consistency with the Funding Information provided in the submission form. The funders and their order are now identical in both sections.

**2) Note: If the reviewer comments include a recommendation to cite specific previously published works, please review and evaluate these publications to determine whether they are relevant and should be cited. There is no requirement to cite these works unless the editor has indicated otherwise.**

We thank the reviewers for their suggestions. We have carefully evaluated the recommended references and included those that were relevant to the scope and context of our study.

**3) Figure resubmission:**

**While revising your submission, we strongly recommend that you use PLOS’s NAAS tool (**[**https://ngplosjournals.pagemajik.ai/artanalysis**](https://ngplosjournals.pagemajik.ai/artanalysis)**) to test your figure files. NAAS can convert your figure files to the TIFF file type and meet basic requirements (such as print size, resolution), or provide you with a report on issues that do not meet our requirements and that NAAS cannot fix.**

**After uploading your figures to PLOS’s NAAS tool -** [**https://ngplosjournals.pagemajik.ai/artanalysis**](https://ngplosjournals.pagemajik.ai/artanalysis)**, NAAS will process the files provided and display the results in the "Uploaded Files" section of the page as the processing is complete. If the uploaded figures meet our requirements (or NAAS is able to fix the files to meet our requirements), the figure will be marked as "fixed" above. If NAAS is unable to fix the files, a red "failed" label will appear above. When NAAS has confirmed that the figure files meet our requirements, please download the file via the download option, and include these NAAS processed figure files when submitting your revised manuscript.**

We thank the journal for this recommendation. All figure files were uploaded to the PLOS NAAS tool and processed accordingly. The figures met the journal requirements, and the NAAS-processed files have been downloaded and included in the revised submission.

**Reproducibility:
To enhance the reproducibility of your results, we recommend that authors of applicable studies deposit laboratory protocols in** [**protocols.io**](http://protocols.io/)**, where a protocol can be assigned its own identifier (DOI) such that it can be cited independently in the future. Additionally, PLOS ONE offers an option to publish peer-reviewed clinical study protocols. Read more information on sharing protocols at** [**https://plos.org/protocols?utm_medium=editorial-email&utm_source=authorletters&utm_campaign=protocols**](https://plos.org/protocols?utm_medium=editorial-email&utm_source=authorletters&utm_campaign=protocols)

We thank the journal for this suggestion. At this stage, the study protocols are fully described in the Methods section of the manuscript. We acknowledge the value of protocol sharing platforms and will consider depositing detailed protocols in protocols.io in future studies.
